# Translations from Foreign Exchanges

**Published:** 1883-02

**Authors:** 


### Translations from Foreign Exchanges.

By O. STROINSKI, M.D.

A CASE OF SYPHILITIC GUMMA IN THE LIVER.

Marie G., fifteen years of age, is very imperfectly developed, and she has suffered for years from scrofula the consequences thereof having been grave lesions in the pharynx and palate with difficult voice, and partial loss of vision by repeated kerato-conjunctivitis. At last she lived in very poor circumstances. A few months ago, she lost some blood through the vagina, and this was thought to be menstruation. She suffered then from severe pains in the back and abdomen, and she noticed a swelling of the latter which increased rapidly, and she was thought to be pregnant. A physician examined her, and declared her to be seven months pregnant, and he sent her to the women's hospital. On examination, the cervix was found normal and very small, hymen intact, pregnancy excluded. The abdomen was very much distended the subcutaneous veins visibly enlarged, and there was palpable in the abdominal cavity a great amount of fluid (ascitic). The liver was enlarged to such a degree that the lower part could be palpated in the region of the umbilicus. The appetite was diminished, there were colicky pains in the abdomen, and she always suffered from diarrhœa. There was no fever or icterus. The patient was put on a diet of milk, and she seemingly improved, but soon all the symptoms returned with repeated vomiting. An aspiration of three liters of the fluid was made, and it was found to be highly albuminous. After repeated aspirations the patient died. The following autopsy showed the organs of the thorax to be in a healthy state, the peritoneum highly inflamed, the uterus and ovaries very small. The very enlarged liver shows an atrophy of its right lobe which is situated in the vertebral cavity, but the left lobe is hypertrophied and forms the greatest part of the organ. The surface shows a general perihepatitis with adhesions to the neighboring parts. The gall-bladder is atrophied and reduced to the size of a small prune, its cavity being inflamed and containing but a little yellowish mucus; the great biliary ducts are permeable. The sections of the liver show the tissue to be firm and consistent and its aspect resembles that of a cardiac liver, the most remote lobules from the center being yellow and fatty, those of the superficial layer red and highly injected. The capsule of Glisson is very small. On the end of the left lobe, the tissue resembles that of the spleen, and the central fatty spots have irregular and ramified folds. In cutting through the small branches of the left liver, there is found on the posterior margin of the liver on the point where the subhepatic veins unite with the vena cava, a small tumor of fatty and translucent tissue resembling caseous substance. The tumor is a true gumma of the liver, and its cicatrix has enveloped in its retraction the two subhepatic veins; it has caused their absolute obliteration, the vena cava presenting a depression without any permeable orifice. This singular lesion explains the alteration of the liver, and it resembles most vividly the cardiac stasis, the cicatricial retraction having caused a veritable ligature of the two principal subhepatic veins. The other organs, and especially those of the genital apparatus, being in contradiction with symptoms of recent syphilitic infection, it is probable that the infection has been caused by vaccination, or that it is a case of hereditary syphilis.—*France Médicale.*

### SALIVARY FISTULA OF THE CANAL OF STENO WITH A NEW SURGICAL PROCEDURE.

If there is made, from whatever cause, a fistula into the integument of the canal of Steno, there will always be a permanent salivary fistula, with not the slightest tendency to reparation, and only an operation will close the fistula. The number of operations for closing the fistula is very large. Dr. Richelat has invented a new procedure which is very simple and which can be applied in all cases. The following cases will show the results of this new operation. A woman, forty-two years of age, had been shot in the right side of the face, twenty-five years ago. There is now an anchylosis of the temporo-maxillary articulation, the teeth cannot be opened more than one millimeter, the food can be introduced but in small particles, and mastication is impossible. There is also a salivary fistula on the most remote parts of the duct, and of the external surface, of the left masseter. This fistula has undergone several changes. There has been formed an abscess opposite to the fistula which broke into the original fistula and there remained a permanent aperture as a new fistula. A scar of the original fistula is now visible, one opening 3 cm. below that from an absess can be seen, and another opening forwards on the buccinator from which the saliva flows constanly. Considering the anterior orifice of the abscess as the true fistula, the following steps of operations were made: A trocar was plunged into the fistula and an exit was made on the buccal mucous membrane on the point where the canal of Steno regularly runs. A canula was then introduced, so that the end of the canula occupied the anterior margin of the new canal and not entering the fistula which was closed by sutures. Thus the saliva took its regular course into the buccal cavity and the posterior aperture healed of itself. In two months' time the cure was perfected.—*L' Union Médical.*

POTT'S DISEASE, PARAPLEGIA, RAPID SPINAL EPILEPSY.

The patient, a young man twenty-three years of age, noticed first rheumatic pains with a slight fever. Three days afterwards his limbs became paralyzed, and he says that he does not know of any cause of the disease, but that he had to walk a good deal. He had no pains, neither in the chest, back or limbs, but then he noticed that he could not stay on his legs. On entering the hospital the legs are found to be bent on the trunk and the flexor muscles are contracted, so that it is impossible to straighten the legs, except with great force and under severe pains. Micturition is painful and there is constipation. The patient can but slowly move his body on the bed. The movement is specially difficult on the left side and there is apparently spinal epilepsy. There is trouble in sensation, with pains in the limbs and formication. He complains also about pains around the waist, and there are points of complete anæsthesia, especially on the upper part of the limbs. He mistakes the sensations of heat, cold, contact etc. There is a vertebral curvature reaching to the fifth dorsal vertebra, but which the patient has never noticed. He died, after a few days, from complete asphyxia. The autopsy shows intramuscular abscess, near the fifth dorsal vertebra. The body of the latter is entirely destroyed and in its place is a caseous mass which has entered the dura mater. The spinal marrow consist of a blackish substance which is rigid and resistant. This mass is also found in the pia mater of the brain and in the cerebral hemispheres, reaching to the interhemispheric fissure. There had been, doubtless, a pressure on the spinal marrow.—*Progrès Médical.*

A CASE OF SYPHILITIC KERATITIS.

The patient, thirty-four years of age, a very muscular and healthy looking man, has been attacked by syphilis with hard chancre, in the year 1870. The present disease dates four months back. The eye became reddened and there were symptoms of photophobia. Varying pains set in with aggravated disturbance of vision. The palpebral conjunctiva is inflamed, the eyeball is injected especially in the pericranial region. The vessels are enlarged and there are seen vessels invading the cornea. This membrane is opaque and the limits of this opacity are diffuse. The iris of the healthy eye is blue, but that of the left side so far as visible is yellow. The pupil is small and there are probably posterior synechiæ. The diagnosis is syphilitic parenchymatous-irido-keratitis. The patient complained often of headache with gastric symptoms and he collapses after two weeks. In the brain there are found in the left hemisphere two gummata. The diseased eye is put in Mueller's solution for two months. The cornea shows a blackish thickening originating from the anterior chamber, and there are folds running from the periphery to the center. The iris is slightly pigmented and its two zones, the central and the peripheric, show prismatic eminences. On the total discision including the not detached cornea, are noticed: 1. An enlargement of the episclera; 2. A declination of the angle of the iris on the posterior surface of the cornea; 3. The corneal tissue is swelled by a blackish mass and there is ablation of the vitreous membrane. There are three synechiæ on the anterior capsule connecting it with the pupil.—*Lyon Médical.*

THE VENOMOUS APPARATUS OF THE SCORPION.

The venomous apparatus of the scorpion is formed by the last abdominal segment and it is curved, pyriform and highly colored. It has two small ducts from which the poison is ejected.

To understand the structure of the apparatus it is necessary to make several transverse incisions. The venomous glands are two in number. Each gland is placed in a cavity which it fills entirely, and the anterior part of which is formed by striated muscular fibres, which can be moved voluntarily by the animal, pressing on the gland and thus separating the poison. Each gland consists of a tegument and a central cavity, which serves as a reservoir for the poison. The tegument is composed of two layers: 1, A fine layer consisting of cellular tissue and muscular fibers, it presents on its internal region lamella-like prolongations which serve to augment the secreting surface; 2. A cellular layer which is composed of prismatic epithelium lining the internal part of the first named layer and the folds of the second one. These epithelial cells are filled with protoplasma, and they contain in great abundance, fine arrayed granulations characteristic of the poison of the scorpion, and which are especially visible on applying acetic acid. The poison escaped by the rupture of the cells and it is accumulated in the central cavity. The venom of the scorpion is a very active poison and its effects are characterized by two phenomena: 1. That of excitement; 2. That of paralysis. One drop of the poison injected into the cellular tissue of the rabbit kills the animal in time of a few minutes. The probability is that the poison acts on the brain and spinal cord.—*L' Union Médicale.*

### TEMPORARY LOSS OF VISION BY PAPILLITIS IN PREGNANT WOMAN.

The patient, a Mingrelian lady, sixteen years of age, has been married three years, and she is now pregnant the third time. She is a brunette of gracile figure; well nourished, and healthy appearance. Four months ago she complained of headache and nausea. After a while the vision became impaired, and palpitation of the heart troubled her, to such an degree that she could find no rest. There was a sensation of heat from the waist up to the chest; throat and head, and the face became very often intensely reddened, but without sweat. The patient being in the seventh month of pregnancy, danced a great deal and took from three to four bottles of strong wine daily. The day before she became perfectly blind she was at a ball, and danced all night. In the three years of marriage she has been but six months free of the pregnant state, and she suffered all this time from nervous affections (globulus-hystericus, hystero-epileptic attacks, etc.) The conjunctiva is not hyperæmic; the pupils slightly dilated; temperature normal. The ophthalmoscopic examination of the right eye was not possible, on account of pain and reflex symptoms, from the slightest rays of light. The left eye showed the retina in state of hyperæmia and inflammation; the veins grossly enlarged. The treatment consisted of absolute rest in a dark room, abstinence from wine, coffee, etc., and a depletion of blood from the temples. After a few days the headache disappeared, and after a week the patient could read common letters. The patient used spectacles of dark glass and took the usual rides in the carriage. The case has to be classed among the neuroses caused, probably, by the repeated cessation of menstruation in such a young body (or some flexion of the uterus), with irregularities in diet. —*Memorabilien.*

### THE ETIOLOGY OF HÆMORRHAGIC VARIOLA.

Last year four Esquimaux died in Paris from hæmorrhagic variola. These patients were young and strong, and not addicted to alcoholism. The autopsy showed the liver enormously enlarged and in complete fatty degeneration; the mesenteric ganglia very developed, the kidneys of extreme volume, fattily degenerated, and the heart in the same condition. It was then believed that the nutrition of these persons, consisting mostly of oil and fish, had aggravated the symptons of the variola. It is a fact well known to surgeons that fatty degeneration of one of these organs provokes very often hæmorrhage, and it is further known that variola excites these organs to fatty degeneration like scarlatina, icterus, typhus and other pernicious diseases. Hæmorrhagic variola happens to occur mostly in debilitated persons, drinkers or pregnant women where fatty degeneration of one or more organs is always present. In the autopsy of persons who died from hæmorrhagic variola, the liver is always found to be fattily degenerated and icterus is always present. The kidneys are altered in this disease to a higher degree than in any other disease. The visceral alterations are not of the importance ascribed them hitherto. It seems rather probable that these lesions have been present more or less before the disease attacked the patients, and that they are in some cases the cause of the rapid and malignant course of the disease. Sometimes there had preceded other zymotic diseases.—*Union Médicale.*

### TOTAL DEVIATION OF THE STOMACH INTO THE LEFT HYPOCHONDRIAC REGION IN A CASE OF CANCER OF THE PYLORUS.

Marie D., a woman seventy years of age, has always had, as she asserts, an "iron" health, and she has menstruated to sixty years of age. But one year ago she began to suffer from digestive troubles, with diarrhœa and alternating constipation. Three months after she noticed a tumor in the abdomen, and severe pains set in, with entire loss of appetite. The lancinating pains made the administration of morphine necessary. The tumor was situated at the left side of the linea alba, 12 cm. below the false ribs. It was movable, as hard as cartilage, and of the size of an orange. There was no ascites, and there never was vomiting during the entire course of the disease. In the autopsy, the stomach was found to be distended with gas and liquids, and it was considerably deviated. In fact, the great transverse diameter of the stomach is vertical, and the pylorus is situated on the left side, kept there by many adhesions, which fix it to the angle of the transverse colon and the descending colon. The intestinal ansæ agglomerated about the pylorous were formed by the colon transversum and those of the ileum. All the adherent ansæ were atrophied by encircling peritoneal folds. The pylorus was in cancerous degeneration, the liver fatty, the venæ saphenæ and femoral of the right side were obliterated.—*France Médicale.*

### Rheumatic Osteitis.

M. Ladiat insists that there are different lesions from the same constitutional cause. Thus, rheumatism affects the bones, the ligaments and the synovial cavities. Osseous rheumatism, or rheumatic osteitis, is anatomically characterized by a considerable swelling of the bones forming exostoses and stalactites. The periosteum is also thickened in certain cases. The osseous tissue is friable, and it has all the histological phenomena of a new generation of bone; osteoplastic layers arranged regularly on the surface of the meshes of the calcareous material passing to the state of characteristic osseous elements. The vessels are augmented and dilated, the spongy tissue predominates. In this disease opposite bones forming an articulation will be attacked. The head of the tibia for instance will have an enlargement thus protruding from the condyle of the femur. The soft parts are as to healthy movements limited and accompanied by crackings due to formation of stalactites. Certain diseases which have been hitherto classified as arthrites will have to be classified anew. This rheumatism is developed in healthy muscular persons who have never suffered from any other rheumatic disease, and cardiac troubles have been observed in this form of rheumatism.—*Revue Médicale.*

### The Action of Quinine on the Heart.

In typhoid fever, large doses of quinine are applied in every civilized country of the world, and it should therefore appear a necessity to publish everywhere the action (often fatal) of quinine on the heart. Small doses of quinine, given at intervals, produce two different actions on the heart. In the first period, the action of the heart is augmented, its contractions more forcible, and the flow of the arterial blood more violent. In the second period, there is a marked decrease, *i. e.*, the pulsations are more irregular in rhythm and force, and there is a tendency of arresting the heart. There is an inhibitory phenomenon following over-irritation. In large doses, the first phenomenon does not appear, but all the symptoms of the second stage appear, with stupor and collapsus, and suppression of the contractions of the heart. Thus are explained the reported cases of sudden death in typhoid fever treated with large doses of quinine. Besides that, the sulph. of quinine is very seldom pure, but it is mostly mixed with forty to fifty per cent. of the sulph. of cinchonine, which has a more deleterious effect on the heart than quinine.—*Revue Médicale.*

SARCOMA OF THE TONSILS CURED BY INJECTIONS WITH IODOFORM.

The patient, a man sixty years of age, suffered from sarcoma of the tonsils, with swelling of the glands of the neck. Billroth and others declined to operate, whereupon, Weinlechner of Vienna, ordered the injections with iodoform, which cured the patient entirely. The case is as follows: The patient noticed the swelling of the tonsils in the year 1881. He then went to Billroth, who told him that the case was incurable, and that he would not operate on it. He then went to Albert, who first promised to operate, but later refused. He then saw Weinlechner who found a tumor as large as a lemon and ulcerated in the left tonsillar region, and another tumor deeply situated and as large as an orange, in the angle of the lower jaw. He was inclined to operate with the permission to make a resection of the jaw, but which the patient refused. He then sent him to Dr. Lebaum who cured the patient by sixteen injections with iodoform. The injections caused a chronic gastritis and later a catarrh of the lungs, and they had to be made in great intervals. To-day there are but eight scars in the tonsillar region and on the arctus palato glossus.—*Memorabilien.*

THE SECRETING EPITHELIUM OF THE KIDNEYS IN THE BATRACHII.

The published works on general anatomy and histology have not fully demonstrated the structure of the epithelium of the kidneys in man. Dr. Bonillot has made investigations on this epithelium in the batrachii. The tubuli uriniferi of this kidney consists of five distinct segments, but from all these structures the second is the most important, and it corresponds with that of the tortuous tube of the uriniferi. This epithelium is composed of polyhedric cells without any cuticulum, but having a broad band on the free surface. These cells contain granular striated matter, and very fine fibrillæ resembling the inter-cellular net, of Klein. The meshes of this net contain a hyaline substance in the interior, and the above-named band is probably due to a condensation of this substance. On some points it is detached in the form of spheric masses, which will be found in the urine. In the normal state there are but a few of these cells, and they are only active cells.—*France Médicale.*

LEUCOPLAKIA BUCCALIS.

This disease, also called psoriasis of the tongue and buccal mucous membrane is a chronic squamous affection of the dorsum of the tongue and the lips, characterized by white elevated spots and superficial induration of the mucous membrane. It is mostly found in persons having been affected by syphilis of the mouth, or in persons addicted to the use of tobacco. But sometimes it is found in persons neither syphilitic nor smokers, and then there are seen round erythematous spots, looking very red, but, later, blackish, and which undergo desquamation. If the disease has continued for several years, there are large, grayish spots, which become black and are thrown off in lamellæ 3 cm. long. Then there is cicatrization with the new formation of epithelial cells, and the surface of the tongue becomes broken and fissured in different directions. At last the tongue shows several elevated points, which look like the papillæ of the tongue of a cat. These points are confluent, and they form at last the true epithelioma.—*L' Union Médicale.*

FISSURE OF THE ANUS AND RECTUM IN A CHILD.

The child, thirteen months old, and very emaciated, suffered from constipation, with slight hæmorrhages from the rectum, and from convulsions on the left side of the body. The child passed, under severe pains, every eighth or tenth day, a mass of hard fæces, whereupon the child became unconscious. Then a bloody-tinged mucus separated from the rectum, with contractions of the sphincter ani. Three to five days before this event, the child suffered from chorea on the left side of the body. After the passage, these convulsions disappeared, and did not return during the next three or five days. The attacks had begun five months previously. The exploration under chloroform showed a fissure of the anus and rectum seven mm. long and three mm. wide. The fissure was divided and healed by iodoform. The hemichorea never reappeared, and the bowels began to move regularly.— *Wiener Med. Wochenschrift.*

### THE METALLIC TUBES OF LEITER AS REGULATORS OF TEMPERATURE.

To regulate the temperature of a certain part of the body our means are very insufficient. A bladder filled with ice, a cataplasma, etc., have been hitherto applied to produce this effect. Leiter, at Vienna, has manufactured flexible tubes of zinc, which can be adapted to any part of the body. A rubber tube is connected with one orifice of the tube so that the latter may be filled with water of any temperature. From the other orifice the water flows out into a basin. The tubes can be applied to the throat, chest, foot, etc. A thermometer is attached to the rubber tube for the regulation of the temperature. A kerosene lamp is used for heating the water in a vessel which is attached at one side to the rubber tube and at the other to a pitcher with water. The temperature can be kept according to the needs in the especial case.—*Progrès Médical.*

### THE AUTOPSY OF GAMBETTA.

Much is said about the autopsy of Gambetta, and the curiosity of the people has been satisfied by the report of an autopsy. The brain has been weighed, and a report was made of several parts of this decomposing body, the stench of which was so penetrating as to make Paul Bert faint like an old woman. But the *mise-en-scène* is deficient; there is, in fact, no actual official report of an autopsy. The wound was cicatrized; but where was the wound? in the arm, or in the abdomen? In the absence of all other explanations, we are inclined to believe that it was in the latter region, *i. e.*, there was a purulent infiltration of the abdominal gland, and a profound abscess behind the colon. We hope that the enlightened physicians who made this autopsy will throw more light upon this point.—*Revue Médicale.*

PURPURA RHEUMATICA.

J. F., thirty-seven years of age, suffered from rheumatic pains in the knee-joints, the arms and the chest. Swelling around the joints appeared, but when this ceased, purpura appeared on the lower limbs. The gums bled easily. Then the joints of the right arm began to swell, and the purpura appeared here. After five days, the joints of the left arm began to swell, and there was immediately an eruption of purpura. The eruption was formed by hæmorrhagic spots, which, when opened, discharged a bloody fluid. Black spots remained for a long time.—*Med. Chir. Centralblatt.*

THE WEIGHT OF THE BRAIN IN MENTAL DISEASES.

Dr. Bra has made a series of investigations on this point in the Asylum of St. Anne. The result, he says, is that in mental diseases the brain weighs a little more than usual, and the circumference of the cranium is also a little larger. But the weight is changing with the intellect which the person possessed. The brain of epileptics does not weigh more than that of a healthy person. In mental diseases, there is always a difference in the weight between that of the right and that of the left lobe.—*France Médicale.*

The *Progrès Médical*, speaking in its student number about the colleges of the different countries, says that most of the colleges in the United States could be closed to the advantage of the people. This is rather rough on us, and we have but one consolation, *i. e.*, that the average French medical college, except that of Paris, gives no better opportunity to the medical student than those of our country. The better colleges of the American College Association can well compare with those of France and Italy.

DR. CLEMENCEAU, the admirable leader of the left wing in the French Assembly, has a good practice in Paris. Sitting one day in his office busily engaged with the registration of the patients whom he had prescribed for, a young man entered the office and the doctor ordered him to take off his coat. The young man took off all his clothes, and the doctor, looking suddenly at the nude figure of the strong and healthy man cried out: "What in heaven's name do you want?" "I would ask you for a position in the post office," the young man replied. Tableau.

THE ACTION OF STRYCHNINE ON DILATATION OF THE HEART.

Patients who had a well stated dilatation of the heart took strychnine daily, and the diameter of the heart was measured daily. The results are as follows: 1. After one or two days, the enlargement decreased so that considerable enlargement disappeared. 2. If the use of strychnine is suddenly stopped, the enlargement will reappear. 3. From two to three mmgrm. sulphite of strychnine daily are sufficient to produce these results. —*Wiener Med. Wochenschrift.*

ELECTRICAL TREATMENT OF EPIGASTRIC PAINS IN HYSTERIA.

These pains, which are mostly accompanied by severe vomiting, have been successfully treated with the galvanic current by Dr. Apostoli. The positive pole is applied in the subclavicular region and the negative pole over the seat of the pain. It is continued for 5 to 15 minutes and it is said to have stopped the vomiting entirely. The gastralgia and epigastric pains have been stopped after ten to fifteen applications.—*Revista Medico-Quirurgica.*

THERE is a revival of the old box-theory in generation which had been nailed up by Haller two hundred years ago. According to this theory Eve had all the coming generations in her two ovaries incorporated as one box is inclosed in the other.

THE Argentine Republic gives liberal "stipendia" to young physicians who have excelled in their examinations, and they are thus enabled to go abroad and finish their studies in Europe.

